# First report on the molecular detection and genetic diversity of *Anaplasma marginale* in healthy dairy cattle in Khon Kaen province, Thailand

**DOI:** 10.14202/vetworld.2024.389-397

**Published:** 2024-02-16

**Authors:** I Putu Gede Yudhi Arjentinia, Bamphen Keomoungkhoun, Chaiyapas Thamrongyoswittayakul, Somboon Sangmaneedet, Weerapol Taweenan

**Affiliations:** 1 Division of Pathobiology, Faculty of Veterinary Medicine, Khon Kaen University, Khon Kaen 40002 Thailand; 2 Division of Livestock Medicine, Faculty of Veterinary Medicine, Khon Kaen University, Khon Kaen 40002, Thailand

**Keywords:** *Anaplasma marginale*, bovine anaplasmosis, dairy cattle, molecular prevalence

## Abstract

**Background and Aim:**

Bovine anaplasmosis (BA) is one of the most important diseases of ruminants worldwide, causing significant economic losses in the livestock industry due to the high morbidity and mortality in susceptible cattle herds. *Anaplasma marginale* is the main causative agent of BA occurring worldwide in tropical and subtropical regions. This study aimed to investigate the first molecular detection and genetic diversity of *A. marginale* in dairy cattle in Khon Kaen Province, Thailand.

**Materials and Methods:**

Blood samples were collected from 385 lactating cows from 40 dairy farms in five districts of Khon Kaen, regardless of age and health status. To detect *A. marginale*, all DNA preparations were used for molecular diagnosis using a single polymerase chain reaction with the *msp4* gene target. A phylogenetic tree was constructed from the *msp4* gene sequences using molecular genetic characterization. Genetic diversity was calculated as haplotype diversity, haplotype number, number of nucleotide differences, nucleotide diversity, and average number of nucleotide differences.

**Results:**

The overall prevalence of *A. marginale* was 12.72% (49/385). The highest prevalence (17.19%) was found in Ubolratana district, followed by Muang, Kranuan, Khao Suan Kwang, and Nam Phong districts (14.94%, 14.74%, 13.79%, and 3.70%, respectively). Phylogenetic analysis showed that *A. marginale* was closely related to isolates from Australia (98.96%), China (99.68%), Spain (99.74%), and the USA (99.63%).

**Conclusion:**

The molecular prevalence of BA in dairy cattle is the first to be observed in this area, and the genetic variability with separated clusters shown in the *msp4* gene of *A. marginale* revealed species variation in dairy cattle. This significant genetic diversity contributes to the understanding of the diversity of *A. marginale* and will be important for the control and prevention of *A. marginale* in dairy cattle.

## Introduction

Bovine anaplasmosis (BA) caused by bacteria of the genus *Anaplasma* (*Rickettsiales*: *Anaplasmataceae*) is one of the most prevalent tick-borne diseases worldwide, particularly in tropical and subtropical countries. The Office International des Epizooties Animal Health Code classifies anaplasmosis as a notifiable disease due to economic losses associated with low weight gain, low milk production, abortion, the cost of anaplasmosis treatments, mortality, and its threat to human health [1, 2]. *Anaplasma* species are obligatory intracellular Gram-negative bacteria infecting mammalian blood cells; however, they show different host preferences and cell tropism [3]. BA caused by *Anaplasma marginale* and *Anaplasma centrale* infects and reproduces within cattle red blood cells [4, 5]. *Anaplasma bovis*, found in cattle and buffaloes, infects circulating monocytes [6] and has been reported in humans [7], whereas *Anaplasma ovis* invades and propagates within the erythrocytes of small ruminants, such as sheep and goats [8, 9]. *Anaplasma phagocytophilum*, an emerging human pathogen of public health significance, infects human and animal neutrophils and causes disease in various small mammals and wild animals [8, 10]. Anaplasmosis is biologically transmitted by ticks and mechanically transmitted by biting flies or blood-contaminating equipment [11]. Transplacental transmission from an infected dam to fetuses has also been reported mainly during the second or third trimester, which may subsequently lead to abortion [1, 12, 13].

Detection of *A. marginale* infection in cattle includes both direct and indirect methods. It is commonly used to identify the organism in the peripheral blood smear under a light microscope. It is suitable for clinically ill cattle, usually during the acute phase of the disease, but parasites are rarely detected microscopically in chronic infection. Polymerase chain reaction (PCR) is a molecular technique that detects organism DNA with high sensitivity and specificity, particularly in persistently infected cattle [14–16].

A series of surveys on BA using both conventional and molecular methods have been conducted in beef cattle, buffaloes, and ticks from different parts of Thailand [17–23]. However, the molecular epidemiology of BA in dairy cattle, particularly in northeast Thailand, has not been established.

The present study aimed to investigate the molecular detection and genetic diversity of *A. marginale* in dairy cattle in several dairy farms in Khon Kaen province, Thailand. This report also presents a phylogenetic relationship and haplotype diversity (Hd) among the sample isolates from different countries using a phylogenetic tree. The results are expected to provide detailed information on the genetic structure of *A. marginale* and policy-making in the management of *A. marginale* to improve dairy production.

## Materials and Methods

### Ethical approval

This study was approved by the Institutional Animal Care and Use Committee of Khon Kaen University, with recorded number IACUC-KKU-127/64 and reference number 660201.2.11/656 (122). Standard procedures were followed during the collection of blood samples.

### Study period and location

**Figure-1:**
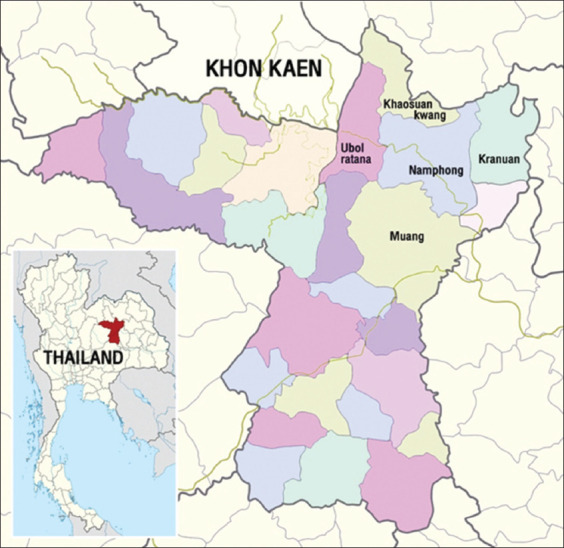
Locations of the research area in Khon Kaen province comprising five districts (adapted from sources: https://en.wikipedia.org/wiki/Khon_Kaen_province and https://fr.m.wikipedia.org/wiki/Fichier: Thailand_Khon_Kaen_location_map.svg).

The study was conducted from July 2020 to October 2021 on smallholder dairy cattle farms located in five districts of Khon Kaen province, including Muang (MN), Kranuan (KR), Nam Phong (NP), Ubolratana (UR), and Khao Suan Kuang (KS) districts (Figure-1). Blood samples from dairy cattle were collected from 40 dairy farms and processed at the Division of Pathobiology Laboratory and Veterinary Diagnostic Laboratory, Faculty of Veterinary Medicine, Khon Kaen University.

### Sample size and sample collection

In view of the lack of data on *A. marginale* in dairy cattle, an estimated prevalence of 50% was assumed. We determined the sample size using the systematic random sampling method, where the minimum number of animals had an absolute precision of 5% and 95% confidence intervals (CI) as indicated by the following formula:


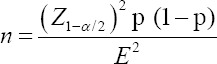


Z=Confidence coefficient (Z = 1.96), n=Sample size, p=Expected prevalence (51%) and E^2^=Absolute precision (5%).

A total of 385 apparently healthy lactating dairy cattle, regardless of age and health status, were included. All dairy cows in the studied areas were crossbred Holstein Friesian and had never been reported to have *Anaplasma* infection before. In addition, there were no ticks on the bodies. Approximately 5 mL of blood sample was collected from the caudal vein of each cattle and transferred to sterile K2 ethylenediaminetetraacetic acid vacutainer tubes (Nipro, Shanghai Co., Ltd., Shanghai, China). Blood samples were stored at −4°C until DNA extraction was conducted.

### DNA extraction

Genomic DNA was extracted from 200 μL of blood using a commercial spin column-based extraction kit (GF-1 blood DNA Extraction Kit, Vivantis Technologies, Malaysia) according to the manufacturer’s protocol. Briefly, 200 µL of each blood sample was transferred into a 1.5 mL microcentrifuge tube, 200 µL of buffer BB was added, and 20 µL of protease K was added and immediately mixed. After incubation at 65°C for 10 min, 200 µL of absolute ethanol was added, and the sample was transferred to a DNA column for centrifugation at 5000× *g* for 1 min. The sample was then discarded. Furthermore, 500 µL of wash buffer I was added to the column, centrifuged at 5000× *g* for 1 min, and discarded. Subsequently, 500 µL of wash buffer II was added to the column, centrifuged at 5000× *g* for 1 min, rewashed with 500 µL of wash buffer II, and centrifuged at maximum speed for 3 min. Finally, 100 µL of preheated elution buffer was added to the DNA sample and stored at −20°C until use. The purity and concentration of DNA were determined using Biodrop (DKSH, UK).

### PCR for *A. marginale*

The *A. marginale msp4* gene was amplified by PCR assay with MSP45 and MSP43 primers [24, 25] (Table-1). The final volume (20 µL) comprised 10 µL of Master Mix (Thermo Fisher Scientific Inc., USA), 1 µL of each forward and reverse primer, 2 µL DNA template, and 6 µL of nuclease-free water. The amplification cycles included initial pre-denaturation at 95°C for 1 min, 35 cycles of denaturation at 94°C for 1 min, annealing at 60°C for 1 min, and extension at 72°C for 1 min, followed by a post-extension at 72°C for 3 min to amplify 840–860 bp target. Agarose gel electrophoresis was also performed.

**Table-1:** List of primers used for the amplification of *Anaplasma marginale msp4* gene.

Primers	Oligonucleotide sequence (5’–3’)	Amplicon size (bp)
MSP45	GGGAGCTCCTATGAATTACAGAGAATTGTTTAC	840–860
MSP43	CCGGATCCTTAGCTGAACAGGAATCTTGC	

### Sequence analysis

Positive amplicons from blood samples were sequenced using BTSeq™ (CELEMICS, Seoul, South Korea). Sequences were compared to sequences reported from *Anaplasma* spp. using the Basic Local Alignment Search Tool (BLAST) of the US National Center for Biotechnology Information (NCBI) (https://blast.ncbi.nlm.nih.gov/Blast.cgi).

### Phylogenetic analysis

Phylogenetic trees were constructed from *msp4* sequences obtained in this study and corresponding sequences from other regions that were banked in genetic databases. Multiple alignment sequences were performed for each locus using the ClustalW algorithm (Conway Institute, University College Dublin, Dublin, Ireland), and genetic relatedness was constructed by the neighbor-joining tree using MEGA version 11 software (Molecular Evolutionary Genetics Analysis, available at https://www.megasoftware.net/). A bootstrap test with 1000 replicates was used to estimate the confidence of the tree branching pattern. The evolutionary distance and similarity distance were computed using the maximum composite likelihood and pairwise distance methods, respectively.

### Statistical analysis of Hd and population characteristics

Hd, haplotype number (Hn), number of nucleotide differences (n), and nucleotide diversity (π) were calculated from the *msp*4 gene sequence alignment using DnaSP (DNA Sequence Polymorphism) version 6.12 software (Universitat de Barcelona, Spain) [26]. The population analysis was performed using TCS (Templeton-Crandall-Sing) Network Analysis version 10.2 software (Computational Science Laboratory, Brigham Young University, USA) with the population analysis Reticulate Trees program (PopART; Population Analysis with Reticulate Trees, University of Otago Popart, New Zealand) [27]. Genetic differentiation and estimate pairwise statistics among *A. marginale* isolates based on the *msp4* gene were calculated worldwide using hierarchical analysis of molecular variance (AMOVA) using Arlequin version 3.5 software (Swiss Institute of Bioinformatics, Switzerland) [28, 29]. This study was conducted at the spatial level to evaluate the genetic variability among and within populations.

## Results

### Prevalence of *A. marginale*

**Figure-2:**
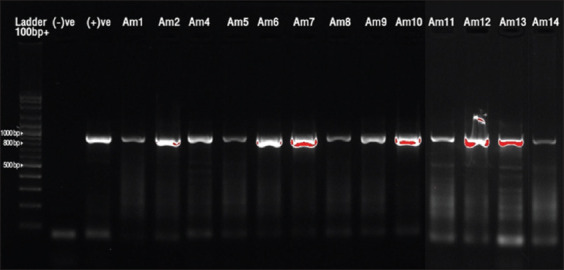
Gel electrophoresis results of the positive bands (860 bp) of *Anaplasma marginale*. Lane Am indicate positive samples, lane ladder with 100 bp plus, lane (−)ve: Negative control, and lane (+)ve: Positive control.

The PCR assay showed that *A. marginale* was detected and identified using the specific primers. The electrophoresis results showed positive samples (860 bp) (Figure-2). *A. marginale* infection was detected in 49/385 samples collected from 40 farms, with an overall prevalence of 12.73% (95% CI = 0.56–1.82). The highest prevalence (17.19%) (11/64) was found in the UR district, followed by the MN, KR, KS, and NP districts (14.94% (13/87), 14.74% (14/95), 13.79% (8/58), and 3.70% (3/81), respectively (Table-2).

**Table-2:** Prevalence of *Anaplasma marginale* from 40 smallholder dairy farms in Khon Kaen province.

District	No. of samples		Standard deviation	95% Confidence interval

	No. tested	No. positive (%)		
Muang	87	13 (14.94)	0.36	0.07–0.23
Kranuan	95	14 (14.73)	0.36	0.07–0.22
Nam Phong	81	3 (3.70)	0.16	0.01–0.06
Ubolratana	64	11 (17.19)	0.39	0.09–0.29
Khao Suan Kwang	58	8 (13.79)	0.35	0.05–0.23
Overall	385	49 (12.72)		

### Phylogenetic analysis

**Figure-3:**
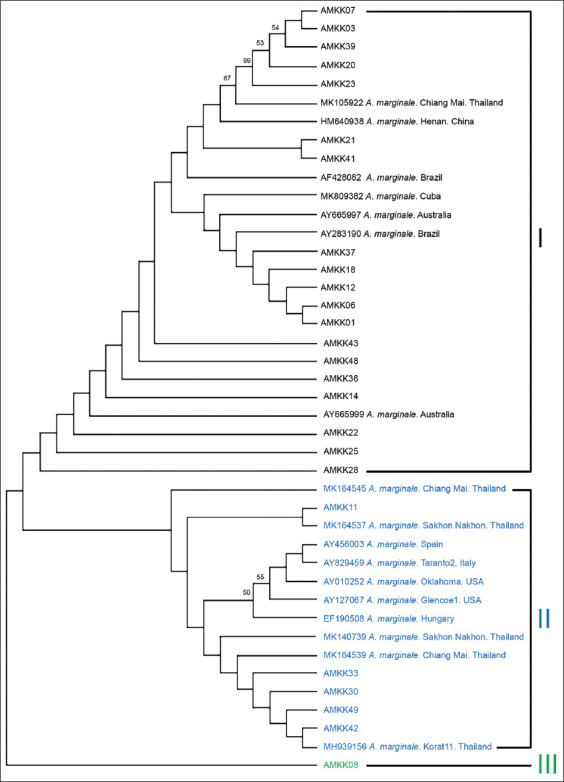
Phylogenetic analysis of local *Anaplasma marginale* *msp4* gene sequences. Phylogenetic tree was constructed using the neighbor-joining method with bootstrap analysis of 1000 replicates (only percentages >50% were represented). The 25 new sequences of *Anaplasma marginale* obtained in the present study are defined with the AMKK (*Anaplasma marginale* Khon Kaen isolate) letter codes. The country of origin and the GenBank accession number are indicated.

Phylogenetic trees were constructed from 25 *msp4* sequences obtained in this study, with 17 sequences obtained from the NCBI GenBank (other parts of Thailand and worldwide) representing the genetic variability of *A. marginale*. The phylogenetic analysis revealed that the *msp4* gene sequences in this study were divided into three clusters (Figure-3). Interestingly, the sequence assigned to the third cluster, separately from the others, showed phylogenetic proximity, representing the genetic variability of the *A. marginale* sequence from Khon Kaen province. The first cluster included strains from China (GenBank accession numbers: HM640938), Thailand (GenBank accession numbers: MK105922), Brazil (GenBank accession numbers: AF428082 and AY283190), Australia (GenBank accession numbers: AY665997 and AY665999), and Cuba (GenBank accession numbers: MK809382). The second cluster contained strains from Thailand (GenBank accession numbers: MK164545, MK164537, MK140739, MK164539, and MH939156), Spain (GenBank accession numbers: AY456003), Italy (GenBank accession numbers: AY829459), the USA (GenBank accession numbers: AY010252 and AY127067), and Hungary (GenBank accession numbers: EF190508).

### Genetic diversity and haplotype analysis

**Figure-4:**
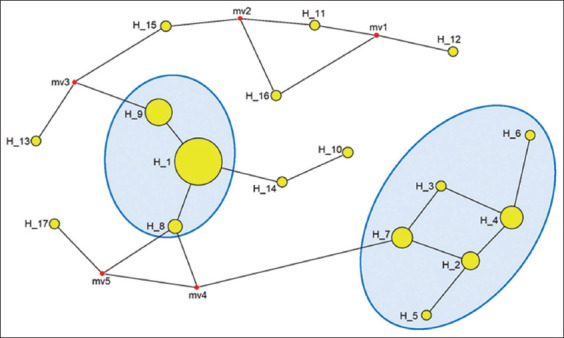
Phylogeographic tree constructed of *Anaplasma marginale* based on *msp4* gene from the local isolates, another part of Thailand, and worldwide haplotypes. Taxon names consist of worldwide haplotypes of the *msp4* gene. Circles with a blue-shaped fill were described as haplotype groups of *A. marginale* isolates from the current study.

A total of 42 *msp4* sequences of *A. marginale* containing 25 isolates were obtained, and 17 isolates from other parts of Thailand and countries worldwide were analyzed using DnaSP version 6.12 software to analyze genetic flow and differentiation and the median-joining network program to construct the genetic divergence and haplotype network. Seventeen haplotype DNA sequences were identified from seven main locations and isolates from five districts of Khon Kaen province (MN, KR, NP, UR, and KS) were obtained within nine haplotype DNA groups (H_1, H_2, H_3, H_4, H_5, H_6, H_7, H_8, and H_9). The PoT (parts of Thailand) group consisted of three haplotype DNA groups (H_1, H_8, and H_9) from another part of Thailand. The last WD (worldwide) group contained eight haplotype DNA groups (H_10, H_11, H_12, H_13, H_14, H_15, H_16, and H_17). The genetic divergence and haplotype network based on the 17 haplotype DNA sequences showed two haplogroups containing isolates obtained in the present study (Figure-4). Haplogroup 1 (H_1) was the dominant haplotype with higher haplotype frequency and was the center of the median-joining network.

The genetic diversity indices differed among populations. Overall, Hn ranged from 2 in NP to 8 in WD with MN, KR, UR, KS, and PoT being 4, 6, 6, 3, and 3, respectively. Hd for MN, KR, NP, UR, KS, PoT, and WD varied from 0.694, 0.844, 1,000, 0.889, 0.800, 0.733, and 0.891, respectively. Nucleotide diversity (p) was lowest (0.001) and highest (0.322) in NP and UR, respectively. Nucleotide diversity in MN, KR, NP, KS, PoT, and WD was 0.322, 0.322, 0.001, 0.231, 0.191, and 0.008, respectively, and the average number of nucleotide differences was 22.100, 22.104, 1.000, 22.122, 15.900, 13.127, and 5.709, respectively (Table-3).

**Table-3:** Genetic diversity indices and test of selective neutrality of *Anaplasma marginale msp4* isolates when compared to another part of Thailand and worldwide sequences.

Location	n	Hn	Hd	π	K	Neutrality test	

						Tajima’s D	Fu’s Fs
Muang	9	4	0.694	0.322	22.100	2.655 (p = 0.99)	21.413 (p = 1.00)
Kranuan	10	6	0.844	0.322	22.104	2.822 (p = 1.00)	15.128 (p = 1.00)
Namphong	2	2	1.000	0.001	1.000	0.000 (p = 1.00)	0.000 (p = 0.24)
Ubolratana	9	6	0.889	0.322	22.122	2.643 (p = 1.00)	12.086 (p = 1.00)
Khao Suan Kuang	5	3	0.800	0.231	15.900	−1.266 (p = 0.99)	11.629 (p = 1.00)
PoT	6	3	0.733	0.191	13.127	−1.544 (p = 0.99)	14.346 (p = 1.00)
WD	11	8	0.891	0.008	5.709	−0.733 (p = 0.49)	1.111 (p = 0.24)

N=Number of sequences, Hn=Number of haplotypes, Hd=Haplotype diversity, π=Nucleotide diversity, K=Average number of nucleotide differences

The demographic history of the population was calculated using the neutrality test based on Tajima’s D and Fu’s Fs. Tajima’s D values for KS, PoT, and WD were 1.266, 1.544, and 0.733, respectively; however, these values were not significantly different in all groups. Fu’s Fs values were not significant in all groups (p > 0.1) (Table-3).

Analysis to determine AMOVA revealed that the greatest genetic variance was observed among populations (34.67%), followed by comparing within populations, and the fixation index (F_ST_) (structure populations/F_ST_) was 65.33%. The F_ST_ value among all isolates was 0.34667 (p < 0.01), implying significant genetic divergence within isolates (Table-4).

**Table-4:** Analysis of molecular variance for the inferred population structure of isolates of *Anaplasma marginale* based on the *msp4* gene in worldwide.

Source of variation	df	Sum of squares	% of variation
Among populations	2	1856.61	34.67
Within populations	70	6425.25	65.33
Total	72	8281.86	

Fixation index (F_ST_): 0.34667 (p < 0.01)

In addition, gene flow (Nm) among the different locations was estimated (Table-5). The Nm value was infinite between UR and KR, KS and MN, KS and UR, and PoT and NP. Distinct gene exchange (17.378) was observed between the UR and NP districts.

**Table-5:** Pairwise differences of F_ST_ values and gene flow (Nm) between isolates of *Anaplasma marginale* based on the *msp4* gene.

F_ST_/Nm	MN	KR	NP	UR	KS	PoT	WD
MN	-	1.196	2.438	2.706	Inf	2.329	2.013
KR	0.111	-	2.588	Inf	6.805	3.084	3.547
NP	0.086	0.166	-	17.38	1.343	Inf	2.939
UR	0.097	0.110	0.248	-	Inf	3.775	1.380
KS	0.037	0.027	0.291	0.088	-	1.973	1.556
PoT	0.180	0.239	0.305	0.298	0.073	-	2.338
WD	0.402	0.452	0.093	0.525	0.156	0.101	-

MN=Muang, KR=Kranuan, NP=Namphong, UR=Ubolratana, KS=Khao Suan Kuang, F_ST_=Fixation index

## Discussion

Anaplasmosis caused by intracellular rickettsia is endemic in several parts of the world and continues to affect dairy production. This disease affects ruminants in Thailand and is an economically important livestock disease. Anaplasmosis has been recorded in cattle in Thailand for more than 35 years since 1986 [30]. The prevalence ranged from 0.03% to 65.2% according to microscopic examination and PCR assay [17].

The prevalence of anaplasmosis in dairy cattle in Khon Kaen province by molecular detection was 12.73% (49/385) with a 95% CI of 0.51–1.94 for *A. marginale* based on the *msp4* gene. In addition to *msp1a*, *msp1b*, *msp2*, and *msp5* [23, 31, 32], the *msp4* gene was used in this study as a good marker for providing sufficient variation to create a large-scale phylogeographic pattern [15]. It is useful for epidemiological analysis of anaplasmosis [33, 34].

The results of this study are similar to those of a previous report by Jirapattharasate *et al*. [17] on beef cattle in Khon Kaen province, with 12% (6/50) prevalence using PCR assay. Other previous epidemiological studies of *A. marginale* in beef cattle were conducted in the northeastern, north, and central parts of Thailand, including 2.4% (2/85) from Mahasarakham, 3.1% (2/65) from Loei, 6.92% (32/462) from Sakon Nakhon, 20% (11/55) from Chiang Rai, 3.3% (2/60) from Mae Hong Son, 58.62% (934/58) from Chiang Mai, 13.51% (25/185) from Nan, 29.84% (57/191) from Nakhon Sawan, and 15.54% (30/194) from Ayutthaya [18, 22, 35].

In an endemic area with no outbreak of anaplasmosis, all positive samples are healthy carrier animals because they do not exhibit any symptoms, remain patent to the vectors, and remain silent sources of infection to other susceptible animals. Good farm management practices, such as vector-borne pathogen control system (insect and tick control), anti-parasitic administration, and clean and disinfected farm equipment, prevent the spread of anaplasmosis. *A. marginale* was detected in 19.08% of *Rhipicephalus microplus* cattle ticks from the upper-northern part of Thailand [36]. The control of vectors should therefore be carefully considered to control BA.

The results of DNA sequence alignment and BLAST analysis confirmed that the phylograms between genotypes had high amino acid content (99.28%). Alignment of several *A. marginale* sequences based on the *msp4* amino acid gene revealed that local isolates contained additional amino acids and specific substitution patterns were unique to isolates obtained from other farms and parts of Thailand. These results indicate that the genetic diversity of *A. marginale* populations varies by geographic region, with additional diversification due to local selection pressure [37]. On the basis of the phylogenetic tree, *A. marginale* was highly conserved in the samples collected in this study and showed a high correlation with other geographical isolates reported previously. Information on the genetic diversity and phylogeny of *A. marginale* strains is limited to the analysis of the *msp4* gene. In the current study, the *msp4* gene was used as a suitable marker to provide sufficient diversity to build a large-scale and useful phylogenetic model for the epidemiological characteristics of *A. marginale* anaplasmosis [37]. Phylogenetic analysis showed that *A. marginale* Khon Kaen isolates could be classified into three groups according to the *msp4* gene. The geographical relationship between isolates cannot be determined because some Khon Kaen sequences are grouped with African, American, European, Australian, and other Asian sequences. Interestingly, one sequence, AMKK48 (*Anaplasma marginale* Khon Kaen isolate 48), differed from the other as a novel genetic variability based on the *msp4* gene from the Khon Kaen isolates.

The *A. marginale* haplotypes were generated with the sequences found in this study and others retrieved from the GenBank database in other regions. The results showed high genetic diversity of the observed *A. marginale msp4* gene compared to previous studies. Similar to previous studies by de la Fuente and Kocan [38], Silva *et al*. [39], and Machado *et al*. [40], independent transmission mechanisms (mechanical and biological) may reflect high genetic diversity in endemic areas. The increase in the number of new circulating genotypes or new strains will lead to greater circulation in nature due to selective pressures and mutations resulting. In endemic areas of *A. marginale*, livestock and ticks that are continuously infested contain heterogeneous isolates of *A. marginale*, indicating that some species forage throughout their life cycle [41]. The genetic heterogeneity of *A. marginale*, resulting in high diversity, can be attributed to various transmission mechanisms, presenting different genotypes and strains in the bovine herd. However, cattle transfers between different geographic regions are a necessary source of dispersion of different isolates of *A. marginale*, which contributes to genetic diversity [42].

F_ST_ values based on haplotype frequency and genetic divergence among the 16S rRNA and *msp4* genes were significant among and within populations. In the current study, most variations of *A. marginale* (83.08%) were found within the populations in terms of the AMOVA analysis results. Thus, this result indicates a significant differentiation between isolate sequences (p = 0.001). Recently, several researchers reported that the F_ST_-value is an important indicator of genetic differentiation between isolates [43]. Low F_ST_-values between isolates indicate high gene flow. The coefficients of genetic differentiation and gene flow between regions indicate that several areas have undergone genetic differentiation in addition to the same region.

The biological and evolutionary significance of the lower genetic diversity of *A. marginale* in dairy cattle in populations near the edge of the species range needs to be further explored using other genetic markers. These markers may provide important insights into the genetic divergence and phylogeographic relationship of *A. marginale* throughout its distributional range. This will have important implications for studies on different types of anaplasmosis-infected pathogens in livestock, domestic animals, wildlife, and humans.

## Conclusion

This study is the first to report the molecular prevalence of BA in dairy cattle in the Khon Kaen province of Thailand. The molecular prevalence of *A. marginale* infection in this area was 12.72%. Genetic variability with separated clusters shown in the *msp4* gene of *A. marginale* revealed species variation in dairy cattle. The PCR method can detect *A. marginale* infection even at low rickettsemia, which is routinely detectable in apparently healthy carrier animals and can serve as a valuable tool under field conditions. In addition, this significant genetic diversity contributes to our understanding of the diversity of *A. marginale*, which will be important for the control and prevention of *A. marginale* in dairy cattle.

## Authors’ Contributions

IPGYA: Data curation, investigation, formal analysis, and writing original manuscript. IPGYA and BK: Sample collection, methodology, and resources. WT, CT, and SS: Resources, supervision, and validation. IPGYA, CT, and SS: Conceptualization, review, and editing manuscript. WT: Conceptualization, funding acquisition, project administration, review, and editing manuscript. All authors have read, reviewed, and approved the final manuscript.
